# Primary bone marrow diffuse large B-cell lymphoma accompanying cold agglutinin disease: A case report with review of the literature

**DOI:** 10.3892/ol.2013.1695

**Published:** 2013-11-21

**Authors:** TOMOKO YAMASHITA, MITSUAKI ISHIDA, HIROKO MORO, HIROFUMI YUMOTO, SACHIKO UCHIBAYASHI, MIYUKI YOSHII, RYOTA NAKANISHI, HIROKO OKUNO, TAKASHI YOSHIDA, TAKAFUMI OKUNO, KEIKO HODOHARA, HIDETOSHI OKABE

**Affiliations:** 1 Department of Clinical Laboratory Medicine, Shiga University of Medical Science, Otsu, Shiga 520-2192, Japan; 2 Division of Blood Service Center, Shiga University of Medical Science, Otsu, Shiga 520-2192, Japan; 3 Division of Diagnostic Pathology, Shiga University of Medical Science, Otsu, Shiga 520-2192, Japan; 4 Department of Hematology, Shiga University of Medical Science, Otsu, Shiga 520-2192, Japan

**Keywords:** diffuse large B cell lymphoma, cold agglutinin disease, primary bone marrow lymphoma

## Abstract

Cold agglutinin disease (CAD) is a well-recognized complication of lymphoproliferative disorders. It has been previously recognized that cases of primary CAD frequently exhibit underlying malignant lymphoma in the bone marrow. Lymphoplasmacytic lymphoma is the most common subtype of malignant lymphoma; however, diffuse large B-cell lymphoma (DLBCL) has also been documented, albeit extremely rare. The current report presents a case of primary bone marrow DLBCL accompanying CAD. A 76-year-old male presented with fever and fatigue. Laboratory tests revealed anemia and elevated bilirubin and cold agglutinins with a titer of 8,192 at 4°C. Bone marrow biopsy demonstrated DLBCL and systemic surveillance failed to detect tumorous lesions or lymphadenopathy. Following R-THP-COP therapy, cold agglutinins titer was markedly decreased (by <4); however, malignant lymphoma relapsed and cold agglutinin levels increased again (4,096). This is the second documented case of primary bone marrow DLBCL accompanying CAD. Previously, malignant lymphoma exclusively involving the bone marrow, namely primary bone marrow lymphoma (PBML), has been recognized as a rare and aggressive subtype. The analyses of the present study revealed that the incidence of hemolytic anemia in primary bone marrow DLBCL may be high compared with conventional DLBCL. Therefore, additional analyses are required to clarify the clinicopathological features of PBML.

## Introduction

Bone marrow involvement by malignant lymphoma is generally considered as a systemic dissemination of the disease arising elsewhere, such as the lymph nodes and extranodal organs. However, albeit extremely rare, malignant lymphomas exclusively involving the bone marrow have been previously reported (1). Recently, the diagnostic criteria for primary bone marrow lymphoma (PBML) have been proposed and the clinicopathological features of this extremely rare tumor have also been documented (1).

Cold agglutinin disease (CAD) accounts for 13–15% of patients with autoimmune hemolytic anemia and is characterized by the presence of cold agglutinins, which are antibodies that agglutinate erythrocytes at an optimum temperature of 0–4°C (2,3). Traditionally, CAD has been classified into a primary type, not associated with malignant lymphoma or other diseases and a secondary type, accompanying infection (such as Epstein-Barr virus and *Mycoplasma pneumoniae*) and malignant disease, most often malignant lymphoma. However, it has been previously recognized that even the primary form is frequently associated with an underlying lymphoproliferative disease in the bone marrow (3). Although 76% of CAD patients have been found to exhibit underlying malignant B-cell lymphoma in the bone marrow (2,3), the incidence of CAD in patients with non-Hodgkin lymphoma is low (4). Previously, Varoczy *et al* reported that of 421 non-Hodgkin lymphoma patients, 7.6% exhibited an autoimmune disease and only one patient exhibited autoimmune hemolytic anemia (5). To the best of our knowledge, only one case of primary bone marrow diffuse large B cell lymphoma (DLBCL) presenting with CAD has been previously reported (6). The present report describes the second case of primary bone marrow DLBCL accompanying CAD. Written informed consent was obtained from the patient.

## Case report

### Case presentation

A 76-year-old male with a past history of traumatic epilepsy presented to Shiga University of Medical Science Hospital (Otsu, Japan) with fever and fatigue. Physical examination revealed jaundice in the patient’s bulbar conjunctiva and no superficial lymph nodes were palpable. The patient did not describe symptoms of acrocyanosis. Laboratory tests demonstrated marked anemia and elevation of total bilirubin and lactate dehydrogenase (hemoglobin, 7.4 g/dl; mean cell volume, 90 fl; white blood cell count, 5.3×10^9^/l; lymphocytes, 2.3×10^9^/l; lactate dehydrogenase, 558 IU/l; bilirubin, 131.7 μmol/l; and soluble interleukin-2 receptor, 1,220 U/ml). Systemic surveillance by imaging studies failed to detect any tumorous lesions, lymphadenopathy or hepatosplenomegaly.

The direct antiglobulin test was positive, with anti-C3d specificity. Anti-IgG was negative and indirect antiglobulin test was also positive. Cold agglutinins were present with a titer of 8,192 at 4°C and <1 at 37°C. Bone marrow aspiration and biopsy were performed.

Subsequently, R-THP-COP (rituximab, 375 mg/m^2^; pirarubicin, 40 mg/m^2^; vincristine, 0.8 mg/m^2^; cyclophosphamide, 650 mg/m^2^; and prednisolone, 40 mg/m^2^) therapy was performed. Following six cycles of R-THP-COP therapy, the cold agglutinin titer was markedly decreased (by <4), and bilirubin (15.6 μmol/l) and lactate dehydrogenase (186 IU/l) levels were also reduced. Bone marrow aspiration revealed no neoplastic lymphocytes. Following 19 months of the initial chemotherapy, malignant lymphoma relapsed. Cold agglutinins were present again with a titer of 4,096 at 4°C. The patient ultimately succumbed to the disease.

### Immunohistochemistry

The formalin-fixed, paraffin-embedded tissue blocks were sectioned (3-μm-thick), deparaffinized and rehydrated. Each section was stained with hematoxylin and eosin and used for immunostaining. Immunohistochemical analyses were performed using an autostainer (Benchmark XT system; Ventana Medical Systems, Inc., Tucson, AZ, USA) according to the manufacturer’s instructions. The following primary antibodies were used: Mouse monoclonal antibodies against bcl-2 (bcl-2/100/D5), bcl-6 (P1F6), CD3 (PS1), CD5 (4C7), CD10 (56C6), CD20 (L26) (all Novocastra Laboratories, Ltd., Newcastle upon Tyne, UK), CD138 (B-A38; Cell Marque Corp., Rocklin, CA, USA) and MUM-1 (MUM1p; DakoCytomation, Glostrup, Denmark), as well as rabbit monoclonal antibody against cyclin D1 (SP4; Nichirei Biosciences Inc., Tokyo, Japan).

### Histopathological findings

**Figure 1:**
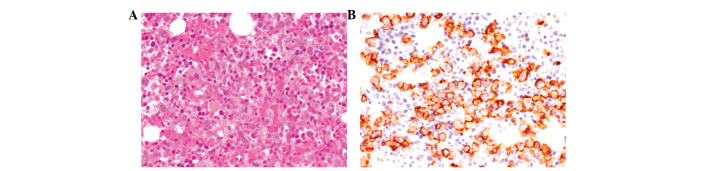
Histopathological and immunohistochemical features of the bone marrow biopsy. (A) Proliferation of large-sized lymphoid cells with large nuclei and conspicuous nucleoli. (B) Lymphoma cells are positive for CD20 (hematoxylin and eosin; magnification, ×400).

Bone marrow aspiration and biopsy revealed hypercellularity with proliferation of large-sized lymphoid cells containing irregular-shaped large nuclei with conspicuous nucleoli (Fig. 1A). No bone trabeculae destruction was noted and no lymphoma cells were detected in the peripheral blood.

### Immunohistochemical findings

Lymphoid cells in the bone marrow were positive for CD20, bcl-2, bcl-6 and MUM1 (Fig. 1B), but negative for CD3, CD5, CD10, CD138 and cyclin D1. According to these results, an ultimate diagnosis of primary bone marrow DLBCL accompanying CAD was concluded.

## Discussion

The present report describes the second documented case of primary bone marrow DLBCL accompanying CAD. Recently, the diagnostic criteria of PBML have been proposed, as follows: i) isolated bone marrow infiltration of lymphoma cells regardless of peripheral blood involvement; ii) no evidence of lymph node, spleen, liver, or other extra bone marrow involvement on physical examination or imaging studies; iii) absence of localized bone tumors; iv) no evidence of bone trabeculae destruction in the bone marrow biopsy; and v) exclusion of leukemia/lymphoma cases (1). The present case corresponded to the abovementioned criteria of PBML. Previously, Martinez *et al* analyzed the clinicopathological features of 21 cases of PBML (1). In total, 15 cases were DLBCL and four were follicular lymphoma. The remaining two cases were peripheral T-cell lymphoma, not otherwise specified. Notably, peripheral blood involvement of lymphoma cells was observed in only one DLBCL case, whereas three of the four follicular lymphoma cases presented peripheral blood involvement (1). No cases of PBML accompanying CAD were identified in the study (1). Níáinle *et al* reported the first documented case of primary bone marrow DLBCL with secondary CAD in a 69-year-old male (6). The patient presented with severe lethargy, and laboratory tests revealed anemia and elevated lactate dehydrogenase and bilirubin. Cold agglutinins were present with a titer of 128 at 4°C and bone marrow biopsy demonstrated DLBCL. The patient was administered R-CHOP therapy, resulting in remission of hemolytic anemia and malignant lymphoma (6). The clinical course of the present case was similar to that of the patient previously reported by Níáinle *et al*(6). Cold agglutinin titer was decreased following chemotherapy in the two cases and increased again at relapse in the present case. These results indicate that lymphoma cells of primary bone marrow DLBCL produce cold agglutinins.

Cold agglutinins in CAD are usually specific for the I antigen, an erythrocyte surface carbohydrate macromolecule. Cooling allows high-thermal amplitude cold agglutinins to bind to the antigen in the peripheral circulation, resulting in the agglutination of erythrocytes and impaired microcirculation. The antigen-antibody complex activates the classical complement pathway, leading to extravascular hemolysis, occurring mainly in the liver (3). The autoantibody in the majority of cases of CAD is IgM-κ (3). Moreover, it has now been recognized that even primary CAD is frequently associated with an underlying lymphoproliferative disease in the bone marrow (3). Previously, Berentsen reported that 76% of primary CAD cases exhibited non-Hodgkin lymphoma in the bone marrow and that the most common type of lymphoma is lymphoplasmacytic lymphoma, followed by marginal zone lymphoma (3). DLBCL accompanying CAD has been extremely rarely reported (4,7). Although, cases of primary pulmonary DLBCL associated with autoimmune hemolytic anemia, primary bone marrow DLBCL complicated with autoimmune hemolytic anemia and erythroid hypoplasia and DLBCL involving the adrenal gland and kidney associated with CAD have also been previously documented (7–9). Primary CAD has now been recognized as a spectrum of clonal lymphoproliferative bone marrow disorder, including DLBCL, as observed in the present case. Therefore, the bone marrow of patients with CAD must be examined for the detection of underlying lymphoproliferative disorders.

Moreover, PBML is an extremely rare and aggressive subtype of malignant lymphoma and <40 cases have been previously reported in the literature (1,6,9,10). In total, three cases of primary bone marrow DLBCL complicated with hemolytic anemia, including the present case, have been documented (6,9). The incidence of hemolytic anemia in this extremely rare type of malignant lymphoma may be high as that of conventional nodal and extranodal DLBCL. Therefore, additional clinicopathological analyses are required to clarify the biological and clinical features of this extremely rare type of tumor.
